# Molecular effects of site-specific phosphate-methylated primer on the structure and motions of Taq DNA polymerase

**DOI:** 10.1016/j.csbj.2023.02.043

**Published:** 2023-02-24

**Authors:** Yi-Chen Tsai, Wen-Yih Chen, Chi-cheng Chiu

**Affiliations:** a Department of Chemical Engineering, National Cheng Kung University, Tainan 701, Taiwan; b Department of Chemical and Materials Engineering, National Central University, Taoyuan 32001, Taiwan; c Hierarchical Green-Energy Materials (Hi-GEM) Research Center, National Cheng Kung University, Tainan 701, Taiwan

**Keywords:** PCR, Primer, Methyl phosphotriester, DNA polymerase, Molecular dynamics

## Abstract

Polymerase chain reaction (PCR) is a powerful molecular biology assay for gene detection and quantification. Conventional DNA primers for PCR often suffer from poor sensitivity in specific gene detection. Recently, oligonucleotides containing methyl phosphotriester (MPTE-DNA) have been developed with enhanced DNA hybridization and improved gene detection sensitivity. Yet, site-specific MPTE-modifications on DNA primers have been reported to affect PCR amplification efficiencies while the detailed mechanism remains elusive. Here, we utilized molecular dynamics (MD) simulation to examine the effects of site-specific MPTE-modified primers on the structure and motions of DNA/Taq polymerase complexes. All tested MPTE-DNA/Taq complexes exhibited RMSD values below 2 Å, indicating insignificant effects of all methylation sites on the complex stability. The energetic and hydrogen-bonding analyses suggest minor effects of methylation at t-3, t-4, t-6, and t-7 positions on the DNA−Taq interaction. Principal component analyses further reveal that only t-3, and t-7 methylations preserve the motions of the Taq thumb domain. The site-specific methylation affects the interaction between DNA and the surrounding protein residues, resulting in allosteric-like effects on the DNA/Taq complex. The MD data complement the best experimentally observed PCR efficacies for the t-3 and t-7 positions among all tested MPTE-primers. The unveiled molecular insights can benefit the design of novel PCR primers for improving genetic testing platforms.

## Graphical Abstract

**Figure:**
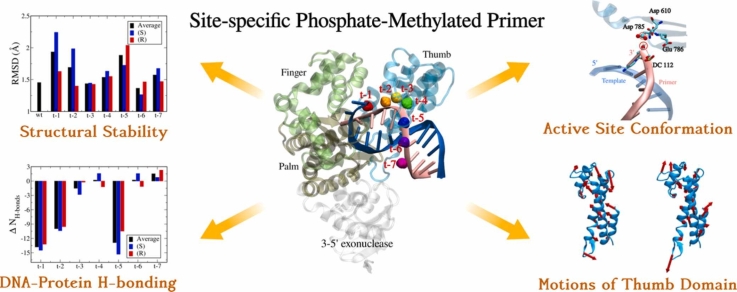

## 1 Introduction

Precision medicine is an innovative medical concept that has attracted increasing attention in recent years [1]. Combining genomic testing, bioinformatics, and big data science, precision medicine creates personalized treatment plans for patients [2], [3]. Among various molecular biology techniques, polymerase chain reaction (PCR) is a sensitive and specific molecular assay widely applied for gene detection and quantification [4]. PCR utilizes DNA polymerase and specific oligonucleotide primers as probes to amplify target DNA for effective gene detection [5], [6]. The specificity and affinity of the primer to the target nucleotide strand are thus critical for detection sensitivity and accuracy.

One of the key components for PCR is the suitable DNA polymerase that can remain active at high temperature required for DNA denaturation. DNA polymerase binds the partial double-helix hybridized from the primer and the template strand and elongates the primer by complementing the template strand to complete the full double-helix [5], [7]. The structure of a DNA polymerase, despite its wide sequence variety, is shaped like a human right hand with three distinct domains, namely the palm, finger, and thumb domains. Each domain plays an important role in catalyzing DNA polymerization. The thumb domain involves the localization, processivity, and transfer of double-stranded DNA. The fingers domain guides crucial interactions between the incoming deoxynucleoside triphosphates (dNTPs) and their corresponding template bases. The palm domain contains the active site of polymerase and catalyzes the phosphoryl transfer reaction [8]. Depending on the type of PCR and the nature of the target DNA, specific polymerases or mixtures may be used for PCR optimization [8], [9].

Note, however, general DNA primers used as probes for target genes often lack specificity and sensitivity to identify short strands or sequences with high CG content [10], [11]. To improve the specificity and sensitivity, many nucleotide derivatives have been developed, such as locked nucleic acid (LNA) or peptide nucleic acid (PNA). LNA is an RNA analogue with a methylene linkage introduced between 2′-oxygen and 4′-carbon in the RNA structure, restraining the LNA nucleotide monomer into 3′ endo conformations [12]. This increases the melting temperature ™ of the LNA-modified primer, corresponding to the high hybridization affinity between the primer and the target strand [13]. LNA has been widely used in microRNA (miRNA) detection [14]. PNA is a DNA analogue with the negatively charged phosphate-deoxyribose backbone replaced by an uncharged N-(2-aminoethyl) glycine unit. Due to the absence of Coulombic repulsion, PNA sequences can bind to their complementary single strand with better thermal stability than DNA-DNA duplexes. Such properties make PNA an ideal candidate as a capture probe for the biosensing field [15].

**Fig. 1:**
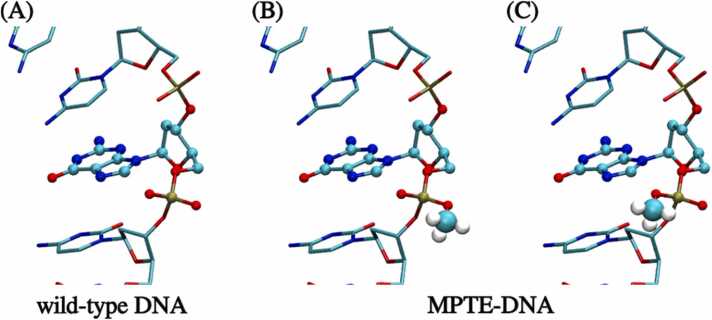
(A) Structure of the wild-type DNA with conventional phosphodiester linkage. (B) S isomer of DNA with a methyl phosphotriester (MPTE) linkage. (C) R isomer of DNA with an MPTE linkage. The target nucleotide residues for MPTE-modification are illustrated in ball-and-stick models with the methyl groups emphasized with vdW representations.

Recently, oligonucleotides with methyl phosphortriester (MPTE) inter-nucleotide linkage(s) have been reported as novel DNA analogues applied in biosensing [16]. As illustrated in Fig. 1, the methylation neutralizes the negatively charged phosphate diester linkage, leading to neutral DNA (nDNA) primer. The MPTE modifications on primers affect PCR efficacies from two perspectives: the hybridization between the primer and the template DNA and the DNA polymerase activity. When using MPTE-modified oligonucleotides for hybridization, the reduced inter-strand electrostatic repulsion results in a more stable double-stranded structure [17], [18], [19]. Further studies revealed that MPTE-modified primers could be applied to amplify target DNA stands with improved sequence selectivity [17]. Yet, primers modified with MPTE can slightly decrease the activity of common DNA polymerases such as Taq and Pfu. And the degree of PCR efficiency reduction varies depending on the position of MPTE modification on the primers. Notably, primers with MPTE-modification at the 4th and the 8th nucleotide from the 3′ end sequence, i.e. the t-3 and t-7 positions, show the least reduction in PCR amplification efficiency [17]. This indicates that the complex structural variations induced by MPTE-primers with different methylation sites may affect the DNA polymerase activity. However, the exact mechanism by which MPTE-modified primers affect PCR amplification efficiency remains unknown.

MPTE-modifications at different locations may differently alter the interactions between the modified nucleotides and the surrounding amino acids, leading to various effects on the structural stability of the DNA/protein complex and thus the PCR amplification efficiencies. Here, we applied molecular dynamics (MD) simulations to examine the molecular interactions between MPTE-modified primers and Taq DNA polymerase. We further utilized principal component analysis (PCA) to characterize the effects of site-specific MPTE-modification on the motions of the protein. The combined results complement the reported experimental data and provide detailed insights into the MTPE-modified primer design.

## 2 Simulation method

**Fig. 2:**
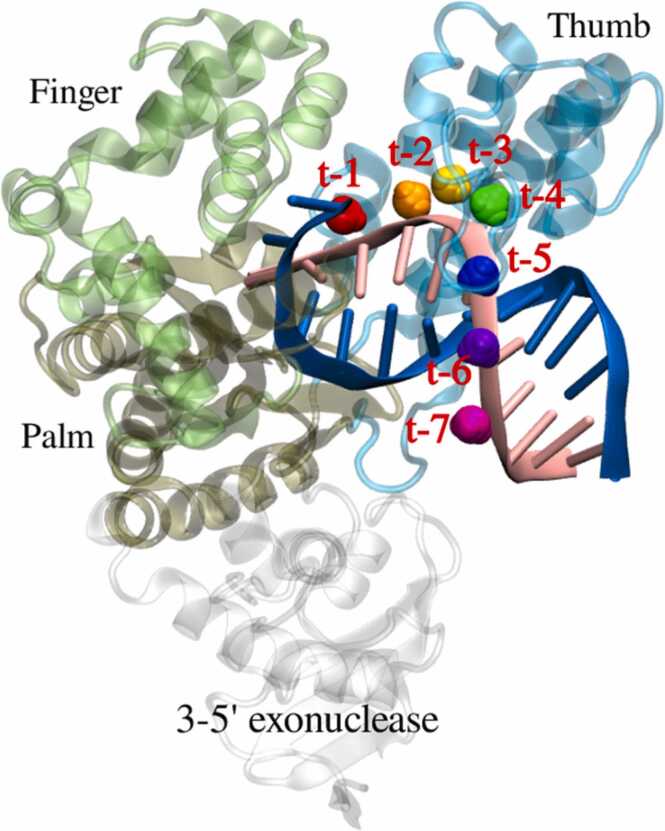
The conformation of the MPTE-DNA/Taq complex. The thumb, finger, palm, and 3–5′ exonuclease domains are displayed in transparent cyan, lime, tan, and gray respectively. While the DNA primer and template strands are painted in pink and blue, and the 7 MPTE-modification positions on the primer are presented with colored spheres.

The conformation of the DNA/Taq complex was taken from the Protein Data Bank with the PDB ID of 4DLG [20]. As shown in Fig. 2, the complex structure contains the Taq polymerase and two DNA segments, including a 12-residue primer and a 16-residue template segment. The Taq structure in 4DLG includes the inactive 3′− 5′ exonuclease domain and the polymerase domain consisting of the three distinct thumb, finger, and palm domains as illustrated in Fig. 2. The sequences of the template DNA, the primer, and the designed site-specific MPTE-modified primers are listed in Table 1, where the “t-n” notation denotes the MPTE-modification at the n + 1th residue from the 3′ end of the sequence.

**Table 1:** PCR template and primers sequence applied in the study. Superscript “n” denotes the residue with the MPTE-modification.

Name		Sequence
Template		3′-CTGGTGCCGCGGGAAA-5′
Primer	wt	5′-GACCACGGCGCC-3′
	(S)-t-1 (R)-t-1	GACCACGGCGC^n^C
	(S)-t-2 (R)-t-2	GACCACGGCG^n^CC
	(S)-t-3 (R)-t-3	GACCACGGC^n^CGC
	(S)-t-4 (R)-t-4	GACCACGG^n^CGCC
	(S)-t-5 (R)-t-5	GACCACG^n^GCGCC
	(S)-t-6 (R)-t-6	GACCAC^n^GGCGCC
	(S)-t-7 (R)-t-7	GACCA^n^CGGCGCC

For a phosphodiester linkage, there are two oxygens available for methylation as shown in Fig. 1. Hence, two stereoisomers are possible after methylation, namely the R- and S-MPTE isomers. When hybridizing with the template strand, the methyl group of the S isomer is located closer to the minor groove, while the methyl group of the R isomer is closer to the major groove [21]. Experimentally, the synthesis of MPTE-modified oligonucleotides is not stereoselective, leading to a racemic mixture of MPTE-modified primers. In this work, we simulated both R and S isomers for one MPTE linkage to examine the average effects of MPTE-modified primers on the DNA/Taq complex. The van der Waals (vdW) and bonding parameters of the MPTE group were adapted from the general AMBER force field (GAFF) [22]. The partial charges for the MPTE atoms were parameterized using the restrained electrostatic potential (RESP) method following AMBER charge derivation procedure [22]. The geometry optimization and the electrostatic potential (ESP) charges calculations were performed using the density functional theory (DFT) method at HF/6–31 G* level via the Gaussian 03 package [23]. The partial atomic charges were then obtained by fitting the ESP data using the RESP method via the Antechamber package [22], [24]. Note that the charges of the MPTE linkage may slightly differ for various sequences. To generalize the charges, we averaged the RESP charges of MPTE linkages from various dinucleotides found in the primer sequence of the 4DLG complex, including CC, CG, GC, CA, and AC. Table S1 in Supplementary material lists the resulting charges for the MPTE group.

Each DNA/Taq complex was first immersed in a 10.4 × 10.4 × 10.4 nm^3^ cubic box filled with water molecules and neutralized with sodium ions. The initial configuration was further energy minimized with the steepest descent minimization algorithm to eliminate high-energy conformations. The system was then pre-equilibrated with a 100 ps canonical ensemble (NVT) simulation followed by another 100 ps isothermal-isobaric (NPT) simulation. The production NPT simulation was then conducted for 240 ns, where system configurations were saved every 10 ps during the last 100 ns trajectories for further analyses. Temperature was controlled at 341 K, the optimal temperature of Taq polymerase, using the modified Velocity-rescale coupling with a stochastic term [25]. Pressure was maintained at 1 bar via the Parrinello-Rahman algorithm [26]. The AMBER99SB* -ILDN force field [27], [28] was used to describe the Taq protein and polynucleotide. TIP3P model was applied for water molecules [29]. The vdW and short-range electrostatic potentials were cut-off at 1.0 nm without any switch functions. Long-range electrostatic interactions were evaluated using the Particle mesh Ewald (PME) method [30], [31]. All bonds were constrained at their equilibrium lengths using the LINCS algorithm [32]. All MD simulations were carried out using GROMACS 2016.4 software package with 2 fs integration timestep [33], [34]. System configurations were visualized with visual molecular dynamics (VMD) software [35]. All the analyses were performed by the built-in modules of GROMACS and in-house analysis scripts [33], [34].

To examine the motions of Taq protein, we conducted principal component analysis (PCA) on 128 C_α_ atoms of the Taq thumb domain over the last 100 ns equilibrium MD trajectories for each system. The calculation of principal components (PCs) consists of two steps [36], [37]. A 3 N-dimensional covariance matrix C (N = 128 in this work) is first constructed using the positional deviation of the protein structure. Each element of C is defined as

$$C_{\mathrm{ij}}=\langle \left(x_{i}-\langle x_{i}\rangle )(x_{j}-\langle x_{j}\rangle \right)\rangle$$

where $x_{i}$ and $\langle x_{j}\rangle$represent the instantaneous and the average cartesian coordinates of the i^th^ C_α_ atom, respectively. The matrix C is then diagonalized to calculate the eigenvalue E:

$$E=A^{T}\mathrm{CA},$$

where A is the eigenvector corresponding to an eigenvalue E. The eigenvalues represent the mean-square fluctuations in the direction of the principal mode [38]. And the first component (PC1) corresponding to the largest E value thus represents the protein motion with the largest mean-square fluctuation and contributes most to the overall motions. The conformational changes of protein can thus be characterized by the first few PCs with the largest E values [39], [40]. Here, the calculation of PCA was performed with the GROMACS software package [33], [34]. The resulting PCs were visualized with VMD package [35].

## 3 Results and discussion

### 3.1 DNA/Taq complex stability

**Fig. 3:**
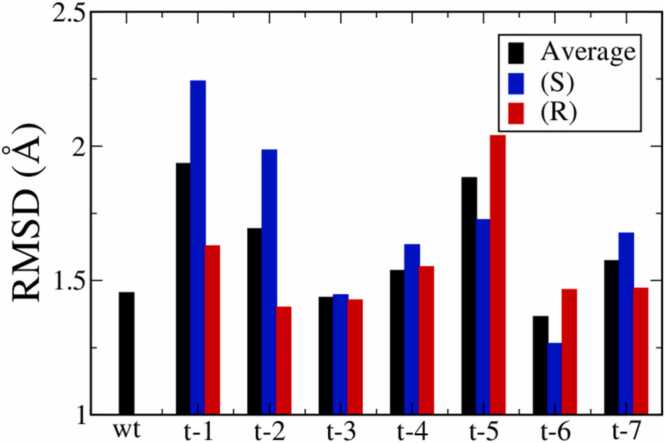
The average RMSD values of wt-DNA/Taq and MPTE-DNA/Taq complexes obtained from 100 ns trajectories after equilibration. The blue and red bars correspond to the results of S-MPTE/Taq and R-MPTE/Taq complexes, respectively; whereas the black bars are the average values of the two isomers.

**Fig. 4:**
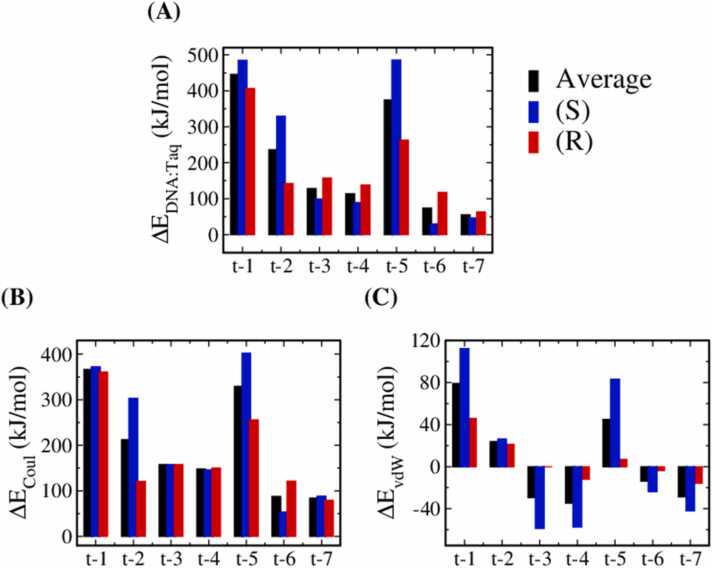
The difference of the nucleotide−Taq interaction ($∆E_{\mathrm{DNA}:\mathrm{Taq}}$) before and after methylation of each MPTE-DNA/Taq complex. (A) The average $∆E_{\mathrm{DNA}:\mathrm{Taq}}$ values and the respective contributions of (B) Coulombic ($∆E_{\mathrm{Coul}}$) and (C) van der Waals interactions ($∆E_{\mathrm{vdW}}$). The blue and red bars correspond to the results of S and R isomers, respectively; while the black data are the average results of the two isomers.

To examine the effects of site-specific MPTE-modified primer on DNA polymerase efficacy, we conducted a series of MD simulations on the DNA/Taq complex system (PDB ID: 4DLG) with various primers methylated at t-1 to t-7 positions, corresponding to the MPTE-modification at 2nd to 8th residues from the 3′ end. Two stereoisomers, S and R, for each methylation site were also studied to evaluate the average effects of MPTE-modification. As illustrated in Figs. 2, t-1 to t-4 MPTEs are located within the Taq thumb domain, t-5 is near the edge of the thumb domain, and t-6 and t-7 are merely in contact with Taq protein. The residues surrounding each methylation position (within 5 Å) are listed in Table 2. The neighboring residues interacting with the MPTE methyl group can be further classified using a 4.5 Å cut-off criteria. In t-3, t-4, t-6, and t-7 primer systems, the methyl groups at the S and R sites have similar neighboring residues. However, in the t-1 and t-2 systems, the neighboring residues at the S site are more than those at the R site, while the trend is reversed in the t-5 primer system.

**Table 2:** Residues surrounding the MPTE linkage and the methyl groups. The superscript “ ^+^ ” and “ ^–^ ” denote the positively and negatively charged residues, respectively.

	Within 5 Å of Phosphate	Within 4.5 Å of Methyl Group
(S)-t-1 (R)-t-1	Pro585, Val586, Arg587^+^, Arg660^+^	Pro585, Arg587^+^ Arg587^+^
(S)-t-2 (R)-t-2	Glu537^–^, Lys540^+^, Arg587^+^	Glu537^–^ -
(S)-t-3 (R)-t-3	Ser515, Ala516, Arg536^+^	- -
(S)-t-4 (R)-t-4	Arg487^+^, Thr506, Ser513, Thr514, Ser515	Ser513, Thr514 Ser513, Ser515
(S)-t-5 (R)-t-5	Thr506, Glu507^–^, Lys508^+^, Thr509	Thr509 Glu507^–^, Lys508^+^
(S)-t-6 (R)-t-6	Lys508^+^	Lys508^+^ Lys508^+^
(S)-t-7 (R)-t-7	-	- -

To characterize the structural stability of the DNA/Taq complex, we first analyzed the root mean square deviation (RMSD) of the complex from MD trajectories. As illustrated in Fig. S1 in Supporting Information, the RMSD values for all tested systems plateaued after 140 ns, suggesting the equilibration of the complex conformations. Fig. 3 displays the average RMSD values over 100 ns trajectories after equilibration for all systems. Comparing RMSD values averaged over S and R isomers, all MPTE-primer systems exhibited RMSD values below 2 Å. Such conservations of DNA/Taq complex conformations can correspond to the experimentally observed PCR activities for all MPTE-primers [17]. Particularly, the RMSD values of t-3, t-4, t-6, and t-7 primers are close to the one of the wild-type (wt) primer, suggesting that methylation at these positions may have minor effects on the MPTE-DNA/Taq complex conformations. Furthermore, the RMSD values for t-1, t-2, and t-5 primer systems exhibit distinct discrepancies for S and R isomers, mainly due to the difference in the contact residues between Taq and the methyl groups. For other positions, there are no significant RMSD differences between the two methylation sites.

### 3.2 Effects of MPTE-modifications on DNA−Taq interactions

To better understand the molecular effects of methylation, we further analyzed the difference of the nucleotide−Taq interaction $∆E_{\mathrm{DNA}:\mathrm{Taq}}$ before and after MPTE-modification:

$$∆E_{\mathrm{DNA}:\mathrm{Taq}}=E_{\mathrm{MPTE}-\mathrm{DNA}:\mathrm{Taq}}-E_{\mathrm{wt}-\mathrm{DNA}:\mathrm{Taq}}.$$

In MD, the DNA−Taq interaction can be further divided into the Coulombic ($E_{\mathrm{Coul}}$) and van der Waals ($E_{\mathrm{vdW}}$) interactions:

$$∆E_{\mathrm{DNA}:\mathrm{Taq}}={∆E}_{\mathrm{vdW}}+{∆E}_{\mathrm{Coul}}.$$

Fig. 4 displays the averaged $∆E_{\mathrm{DNA}:\mathrm{Taq}}$ over the two stereoisomers and the respective contributions of ${∆E}_{\mathrm{vdW}}$ and ${∆E}_{\mathrm{Coul}}$. Higher values indicate greater destabilizing effects of methylation on the DNA/Taq complex. All tested MPTE-primer systems exhibited higher $∆E_{\mathrm{DNA}:\mathrm{Taq}}$ values, suggesting reduced DNA/Taq complex stability. Primers methylated t-3, t-4, t-6, and t-7 positions exhibit smaller $∆E_{\mathrm{DNA}:\mathrm{Taq}}$ variations, indicating reduced destabilization effects of MPTE-modification. Such results are also consistent with the smaller RMSD values for these primer systems shown in Fig. 3.

For all MPTE sites, the magnitudes of ${∆E}_{\mathrm{Coul}}$ are greater than ${∆E}_{\mathrm{vdW}}$, indicating that the methylation mainly affects the electrostatic interaction between the primer and Taq. Note that, positively charged residues located around phosphodiester groups as listed in Table 2 can stabilize the DNA/Taq complex via electrostatic attractions. Methylation neutralizes the negative charge of the phosphodiester linkage and thus reduces the Coulombic attraction between primer and Taq, leading to positive ${∆E}_{\mathrm{Coul}}$ values for all MPTE-primer systems.

In contrast to all positive ${∆E}_{\mathrm{Coul}}$ values, MPTE-modification at different sites leads to divergent ${∆E}_{\mathrm{vdW}}$variations. Specifically, negative ${∆E}_{\mathrm{vdW}}$ are observed in the t-3, t-4, t-6, and t-7 primer systems. This suggests that methyl groups at these positions occupied the void space within DNA/Taq complex and increases the vdW contacts between primer and Taq. Primers with t-1, t-2, and t-5 methylations exhibit positive ${∆E}_{\mathrm{vdW}}$ values, indicating the increased steric repulsions between primer and Taq induced by MPTE-modifications at these sites. Notably, systems with negative ${∆E}_{\mathrm{vdW}}$also have lower ${∆E}_{C\mathrm{oul}}$ values. This suggests that primer methylated at positions with low steric repulsions can help reduce the DNA/Taq complex destabilization effects caused by MPTE-modification.

**Fig. 5:**
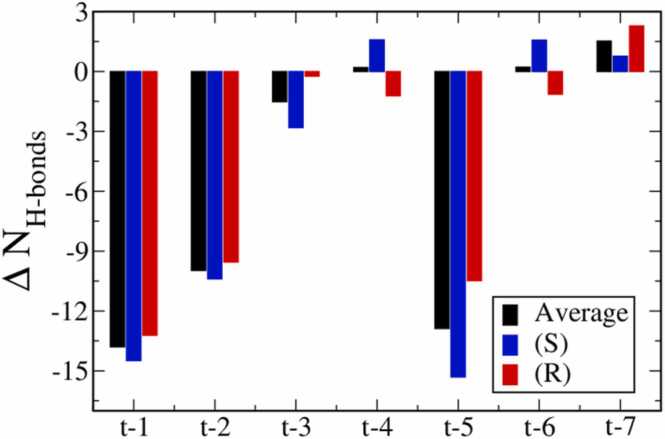
The hydrogen bonds (H-bonds) difference before and after methylation (${∆N}_{H-\mathrm{bonds}}$) for each MPTE-DNA/Taq system. The blue and red bars correspond to the results of S-MPTE/Taq and R-MPTE/Taq complexes, respectively; whereas the black data are the average result of the two isomers.

As illustrated in Fig. 5, we further calculated the difference before and after methylation, ${∆N}_{H-\mathrm{bonds}}$, via averaging the number of hydrogen bonds (H-bonds) formed between nucleotide and Taq over two stereoisomers for each MPTE-primer system. The number of H-bonds for each primer system is shown in Fig. S2 in Supporting Information. In this work, an H-bond was defined topologically with a distance cutoff of 3.5 Å between the donor and the acceptor and an angular cutoff of 30 degrees for the angle of hydrogen-donor-acceptor atoms [41], [42]. For the t-1, t-2, and t-5 primer systems with both S and R isomers, a significant reduction of H-bonds was observed within the DNA/Taq complex. This suggests the destabilization of DNA−Taq interaction after methylation at these sites. In contrast, small ${∆N}_{H-\mathrm{bonds}}$ changes are observed for t-3, t-4, t-6, and t-7 primer systems, indicating the minor effects of these MPTE-modifications on the complex stabilities. The number of H-bonds is slightly increased for either S or R isomers of t-4, t-6, and t-7 primers, owing to the reduced long-ranged electrostatic repulsion allowing the formation of new H-bonds. Note that the ${∆N}_{H-\mathrm{bonds}}$ results are highly correlated to the RMSD and $∆E_{\mathrm{DNA}:\mathrm{Taq}}$ data: a greater reduction of $N_{H-\mathrm{bonds}}$ corresponds to a higher $∆E_{\mathrm{DNA}:\mathrm{Taq}}$ variation and a higher RMSD values. The combined results suggest that t-1, t-2, and t-5 primers have more significant effects on the DNA/Taq complex stability, corresponding to the reduced PCR activities for these primers observed experimentally [17].

### 3.3 Taq catalytic motion analyses

For t-3, t-4, t-6, and t-7 primer systems, MD analyses suggest that these primers have minor structural and energetic effects on the DNA/Taq complex. Yet only t-3 and t-7 primers exhibit the highest PCR efficiencies [17]. This discrepancy suggests that methylation might not only affect the DNA/Taq complex stability but also the conformation and the motion of the active site of DNA polymerase.

**Fig. 6:**
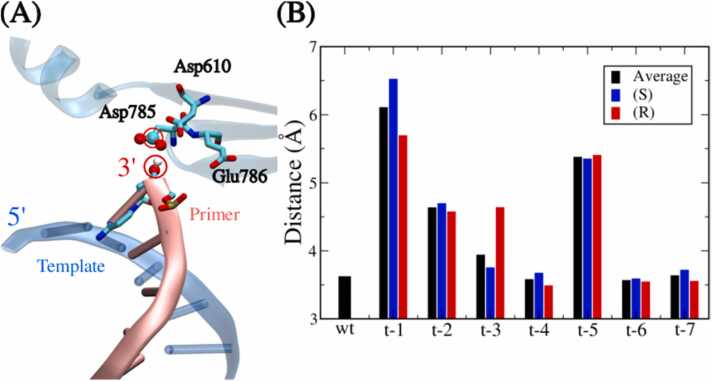
(A) The structures around the Taq active sites. The primer strand is colored in pink, while the template strand and thumb domain of Taq are colored in transparent blue and cyan, respectively. The primer 3′ end and three active site residues are presented in stick model. While the carboxyl group of Asp785 and the hydroxyl group of the 3′ end of the primer are emphasized with a ball-and-stick representation. (B) The average distance between the carbon of Asp785 carboxyl group and the hydroxyl oxygen of the primer 3′ end for each system.

The catalytic site of Taq consists of three carboxylates, i.e. Asp785, Glu786, and Asp610, located close to the 3′ end of the primer as illustrated in Fig. 6 A[43]. To quantify the changes of the active site induced by an MPTE-primer, we measured the average distance between the carbon of Asp785 carboxyl group and the hydroxyl oxygen of the primer 3′ end for each system as summarized in Fig. 6B. The wild-type primer exhibits the closest distance of 3.6 Å, indicating the highest activity of the Taq polymerase. And primers methylated at t-1, t-2, and t-5 positions exhibit significant increased separations, indicating great effects on the active site conformation and reduced Taq activities. The longest distance is observed for the t-1 primer system simply due to the MPTE-modification closest to the active site. Intriguingly, the effects of site-specific MPTE-modification on the active site conformation show similar trends to the effects on the complex structure and stability. This demonstrates the close correlation between the Taq active site and the overall DNA/Taq complex. However, the Taq active site conformation analyses still do not properly rationalize the most PCR efficiency reduction reported for t-4 and t-6 primers.

**Fig. 7:**
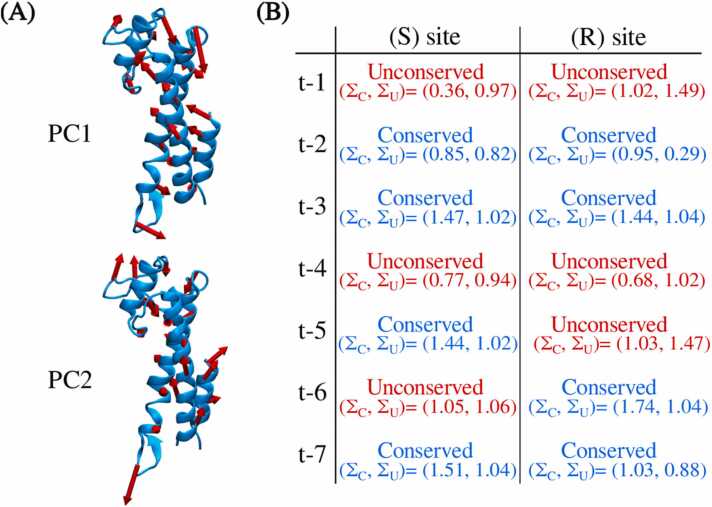
(A) The motions of the Taq thumb domain correspond to the first two principal components (PCs) for the wild-type DNA/Taq system, where the arrows denote the direction and the magnitude of the C_α_ movement for every 128 residues in the thumb domain. (B) The (Σ_C_, Σ_U_) values and the classification of Taq thumb domain motion for both S and R isomers of all tested MPTE-DNA/Taq systems. The red and blue texts emphasize the conserved and the unconserved effects, respectively.

The PCR amplification efficiency is also correlated with the motion pattern of Taq polymerase. The variations in the protein conformation induced by MPTE-primers may also alter the Taq motions and thus its function. Here, we performed principal component analysis (PCA) on each system to characterize the modes of Taq motions. We particularly focused on the motions of the thumb domain, which is in close contact with the primer as illustrated in Fig. 2. The resulting modes of Taq motions corresponding to the two largest principal components (PCs) for the wild-type DNA/Taq system are shown in Fig. 7 A, where the vectors denote the direction and the magnitude of the C_α_ movement. The resulting PCs for all tested MPTE-primers are summarized in Fig. S3 in Supporting Information.

To quantify the effects of MPTE-primer on Taq motions, we took the wild-type primer as a reference and evaluated the directional similarity $\sigma_{\mathrm{mn}}$ between the m^th^ PC of MPTE-DNA/Taq complex and the n^th^ PC of wt-DNA/Taq system as:

$$\sigma_{\mathrm{mn}}=\frac{1}{N}\left[\sum_{1}^{i=N}1-\frac{\theta_{i}^{\mathrm{mn}}}{\pi }\right],$$

$$\theta_{i}^{\mathrm{mn}}=\cos^{-1}\left(\frac{r_{i}^{\mathrm{MPTE}-\mathrm{DNA},m}∙r_{i}^{\mathrm{wt},n}}{\left|r_{i}^{\mathrm{MPTE}-\mathrm{DNA},m}\right|\left|r_{i}^{\mathrm{wt},n}\right|}\right)$$

Here, $r_{i}^{\mathrm{MPTE}-\mathrm{DNA},m}$ represents the eigenvector of the i^th^ atom for the m^th^ PC of MPTE- DNA/Taq complex, $r_{i}^{\mathrm{wt},n}$ denotes the eigenvector of i^th^ atom for the n^th^ PC of wt- DNA/Taq complex, and $\theta_{i}^{\mathrm{mn}}$ is the angle between $r_{i}^{\mathrm{MPTE}-\mathrm{DNA},m}$ and $r_{i}^{\mathrm{wt},n}$. We focused on the main motions of the Taq thumb domain and utilized the first two PCs, i.e., m and n = 1 or 2. $\sigma_{\mathrm{mn}}$ was then obtained via averaging over N = 128 C_α_ atoms in the thumb domain with the maximum value of 1, denoting the identical motion of the MPTE-DNA/Taq complex to the wild-type primer system.

After obtaining the directional similarity $\sigma_{\mathrm{mn}}$ matrix, we further evaluated the motion similarity via summing the diagonal and off-diagonal components:

$$\Sigma_{C}=\sigma_{11}+\sigma_{22}$$

$$\Sigma_{U}=\sigma_{12}+\sigma_{21},$$

where $\Sigma_{C}$ characterizes the conservation of the first two primary PCs and $\Sigma_{U}$ measures the swap of the two PCs. By comparing $\Sigma_{C}$ and $\Sigma_{U}$, we classified the effects of MPTE-primer on the Taq motions into two types: the conserved or the unconserved effect. When $\Sigma_{C}>\Sigma_{U}$, the PC1 and PC2 modes of the MPTE-DNA/Taq complex can correspond to PC1 and PC2 modes of the wild-type primer systems, respectively. In this case, the motion of the thumb domain is conserved, suggesting a minor effect of the methylation on the Taq function. In contrast, if $\Sigma_{C}<\Sigma_{U}$, the PC1 and PC2 modes of the thumb domain motion are altered, leading to unconserved effects of the MPTE-primer on Taq motions and thus its functionality. Fig. 7B summarizes the ($\Sigma_{C}$, $\Sigma_{U}$) values and the resulting classification of Taq thumb domain motion for both S and R isomers of all tested MPTE-primers. The system with conserved effects for both stereoisomers should correspond to better experimental PCR efficiency. Our analysis indicates that only t-2, t-3, and t-7 primers conserve the Taq thumb domain motions. Specifically, t-3 and t-7 systems have larger $\Sigma_{C}$values, whereas t-2 primer leads to the $\Sigma_{C}$ values less than 1. This result indicates that methylation at t-3 and t-7 methylation preserves the motion of the Taq thumb domain and thus the Taq polymerase activity.

### 3.4 Collective effects of site-specific methylation on Taq motions

The energetic and structural analyses demonstrate that methylations at t-3, t-4, t-6, and t-7 positions have minor effects on the DNA/Taq complex conformations. And the t-2, t-3, and t-7 methylations preserve the motions of the Taq thumb domain. Such results can be related to the surrounding residues for each phosphodiester linkage as listed in Table 2. Particularly, there are more than two charged residues near the t-1, t-2, and t-5 positions. Thus, larger variations in RMSD, interaction energy, number of hydrogen bonds, and the active site conformations are observed for the corresponding MTPE-DNA/Taq complexes. For the t-4 methylation, although it has little effect on the complex structure, DNA−Taq interactions, and the active site conformations, it is surrounded by 4 polar residues. The variation in H-bonding patterns thus leads to the changes in the motions of the Taq thumb domain.

**Fig. 8:**
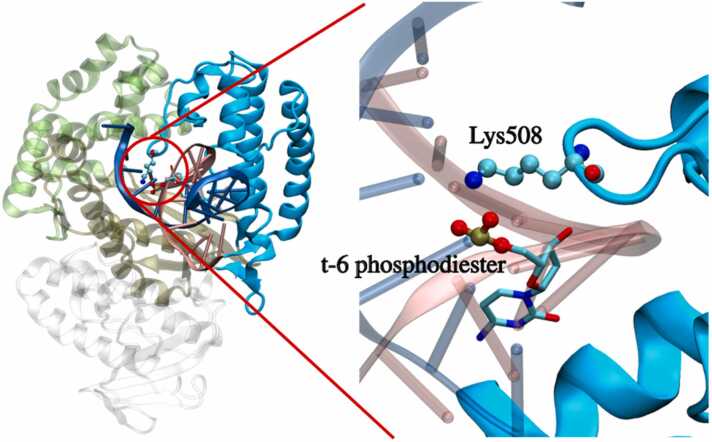
t-6 phosphodiester and its nearby Lys508 residue in the DNA/Taq complex. The t-6 nucleotide is presented in the stick model, and the phosphodiester and the Lys508 residue at the edge of the thumb domain are illustrated in the ball-and-stick representation. The representation of the DNA/Taq complex is identical to Fig. 1, while the thumb domain is highlighted with opaque cyan.

Note, however, the methylation at t-6 position leads to a surprising variation in the thumb domain motions. As shown in Fig. 8, the t-6 phosphodiester is located near the edge of the thumb domain and has one neighboring residue Lys508 on the loop of the thumb domain. The positively charged Lys508 can electrostatically interact with the negatively charged t-6 phosphodiester. Methylation at t-6 position, therefore, alters the correlation between the thumb domain and the primer, leading to the variation of the Taq motion.

**Fig. 9:**
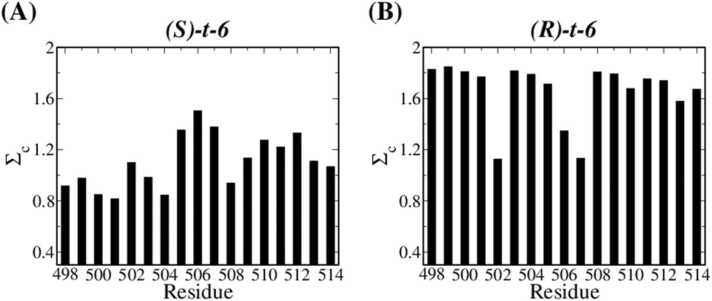
The conservation parameters ($\Sigma_{C}$) for each residue in the loop region of the Taq thumb domain for t-6 primer systems with (A) *S*- and (B) *R*-isomers.

To further characterize the effects of t-6 methylation, we decomposed the contribution of residues in the loop region of t-6 primer systems to the overall conservation parameter $\Sigma_{C}$, as illustrated in Fig. 9. The values are low near the Lys508 residue, particularly for the S isomer where the $\Sigma_{C}$ values are reduced in the entire loop region. This result indicates that the methylation of nucleotide does affect the movement of surrounding protein residues. And the t-6 methylation affects the motion of the loop within the thumb domain, allosterically leading to the unconserved motion shown in Fig. 7.

## 4 Conclusions

In this study, we utilized a series of molecular dynamics simulations to investigate the effects of DNA primer methylated at t-1 to t-7 phosphodiester linkage on the structure and motions of the DNA/Taq polymerase complex. The RMSD analyses showed that methylated primers have little effect on the structural fluctuations of the DNA/Taq complex. Analyses of DNA−Taq interaction, hydrogen bonding, and the Taq active site conformation further suggested the minor effects of MPTE-modification at t-3, t-4, t-6, and t-7 positions on the DNA/Taq complex stability. The PCA analyses suggested that t-2, t-3 and t-7 methylations have minimal effects on the motion of Taq thumb domain, where t-3 and t-7 exhibit the most preservation of the Taq motion. The effects of site-methylations are highly correlated with the number and the type of protein residues surrounding the target phosphodiester groups. Particularly, methylation at t-6 position alters the charges of the phosphodiester linkage, varying its long-range electrostatic interaction with the Lys508 on the loop of the thumb domain. This leads to allosteric-like effects and alters the protein motions with only minor influences on the DNA/complex structure, stability, and active site conformation. The combined results reveal that t-3 and t-7 methylations have minimal effects on the structural stability, DNA-protein interactions, and, more importantly, the DNA/Taq complex protein motions. This complements the experimental observations where the t-3 and t-7 MPTE-DNA primers give the least reduced PCR efficacies among all tested MPTE-primers [17]. The presented results provide invaluable insights into the novel primer design, benefiting the improvement of new generation gene detection platforms.

## Funding sources

This work was partially supported by the Hierarchical Green-Energy Materials (Hi-GEM) Research Center, from The Featured Areas Research Center Program within the framework of the Higher Education Sprout Project by the Ministry of Education (10.13039/100009122MOE) in Taiwan. The authors also acknowledge the financial supports by the National Science and Technology Council of Taiwan (former Ministry of Science and Technology of Taiwan) through Grant Nos. MOST 111-2923-E-006-009- and MOST 111-2221-E-006-006.

## CRediT authorship contribution statement

**Yi-Chen Tsai:** Methodology, Software, Visualization, Investigation, Data curation, Writing – original draft preparation. **Wen-Yih Chen:** Conceptualization, Validation, Supervision, Project administration, Writing – review & editing. **Chi-cheng Chiu:** Conceptualization, Methodology, Software, Validation, Data curation, Supervision, Project administration, Writing – review & editing, Funding acquisition.

## Declaration of Competing Interest

The authors declare that they have no known competing financial interests or personal relationships that could have appeared to influence the work reported in this paper.
