# Supplementary material for: Molecular effects of site-specific phosphate-methylated primer on the structure and motions of Taq DNA polymerase
Source: Comput Struct Biotechnol J. 2023 Feb 24;21:1820–7. doi: 10.1016/j.csbj.2023.02.043 (PMC10009445; doi:10.1016/j.csbj.2023.02.043)
Supplement: Supplementary file 1 — Supplementary material. [file mmc1.pdf]

Molecular Effects of Site-specific Phosphate-Methylated Primer on the Structure and Motions of Taq DNA Polymerase

Supplementary Material

Yi-Chen Tsai[1], *Wen-Yih Chen*[2], *and Chi-cheng Chiu*[1,3,*]

[1] Department of Chemical Engineering, National Cheng Kung University, Tainan 701, Taiwan

[2] Department of Chemical and Materials Engineering, National Central University, Taoyuan 32001, Taiwan

[3] Hierarchical Green-Energy Materials (Hi-GEM) Research Center, National Cheng Kung University, Tainan 701, Taiwan

Table S1. The RESP charges of wild-type DNA and MPTE linkages groups.

Type	Residue	Index	Atom name	Charge
**Wild-type DNA**				
OS	DC/DG	1	O3’	-0.5232
P	DC/DG	2	P	1.1659
O2	DC/DG	3	O1P	-0.7761
O2	DC/DG	4	O2P	-0.7761
OS	DC/DG	5	O5’	-0.4954
CT	DC/DG	6	C5’	-0.0069
H1	DC/DG	7	H5’1	0.0754
H1	DC/DG	8	H5’2	0.0754
**S-MPTE DNA**				
OS	DC1M/DG1M	1	O3’	-0.5232
P	DC1M/DG1M	2	P	1.6185
OS	DC1M/DG1M	3	O1P	-0.5637
O2	DC1M/DG1M	4	O2P	-0.8028
OS	DC1M/DG1M	5	O5’	-0.4954
CT	DC1M/DG1M	6	C1P	0.1673
H1	DC1M/DG1M	7	HC1	0.0648
H1	DC1M/DG1M	8	HC2	0.0648
H1	DC1M/DG1M	9	HC3	0.0648
CT	DC1M/DG1M	10	C5’	-0.0069
H1	DC1M/DG1M	11	H5’1	0.0754
H1	DC1M/DG1M	12	H5’2	0.0754
**R-MPTE DNA**				
OS	DC2M/DG2M	1	O3’	-0.5232
P	DC2M/DG2M	2	P	1.6185

O2	DC2M/DG2M	3	O1P	-0.8028
OS	DC2M/DG2M	4	O2P	-0.5637
OS	DC2M/DG2M	5	O5’	-0.4954
CT	DC2M/DG2M	6	C2P	0.1673
H1	DC2M/DG2M	7	HC1	0.0648
H1	DC2M/DG2M	8	HC2	0.0648
H1	DC2M/DG2M	9	HC3	0.0648
CT	DC2M/DG2M	10	C5’	-0.0069
H1	DC2M/DG2M	11	H5’1	0.0754
H1	DC2M/DG2M	12	H5’2	0.0754

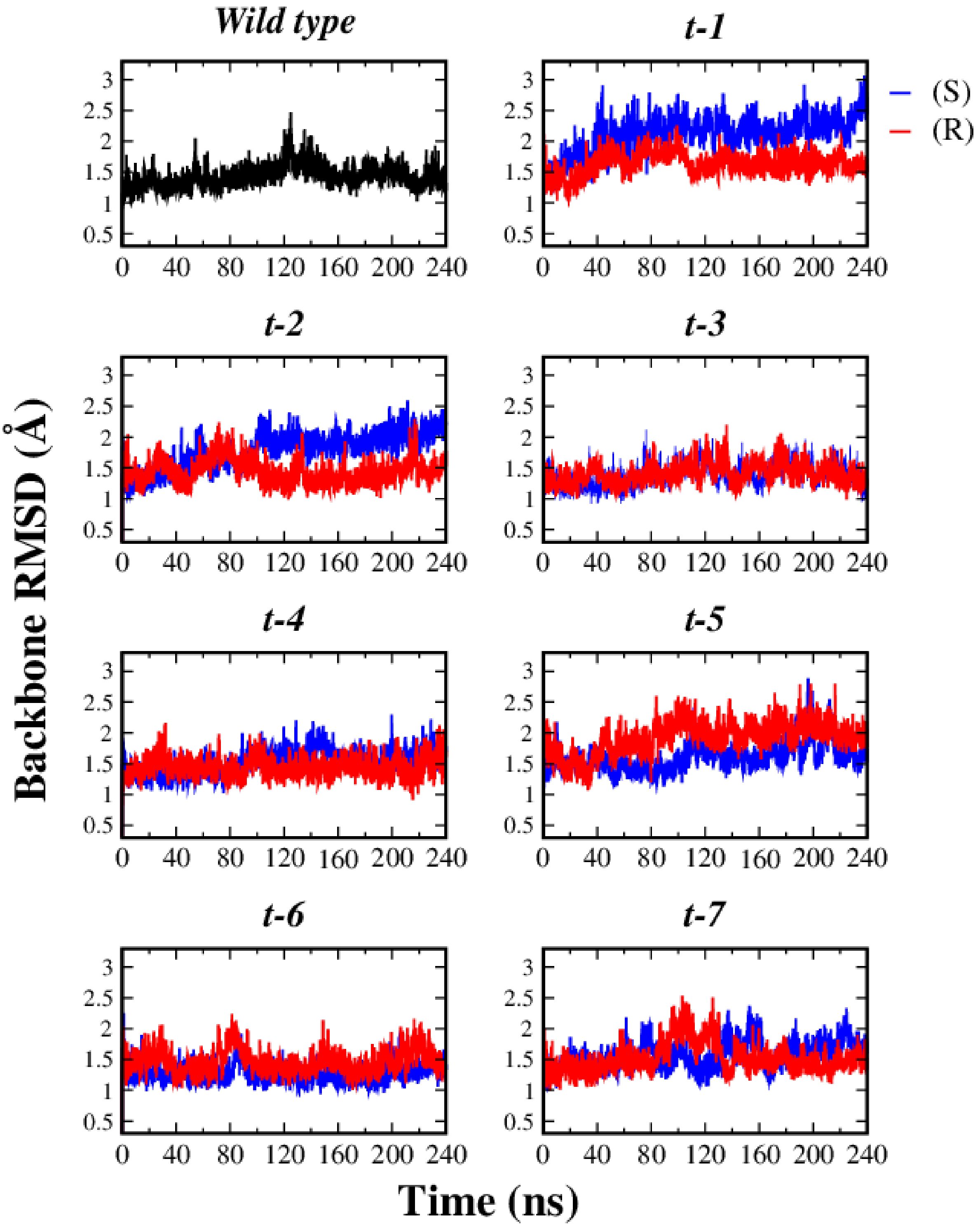

Figure S1. The root mean square deviation (RMSD) of wt-DNA/Taq and MPTE-DNA/Taq complexes backbone from MD trajectories. The blue and red data correspond to the results of S-MPTE/Taq and R-MPTE/Taq complexes, respectively.

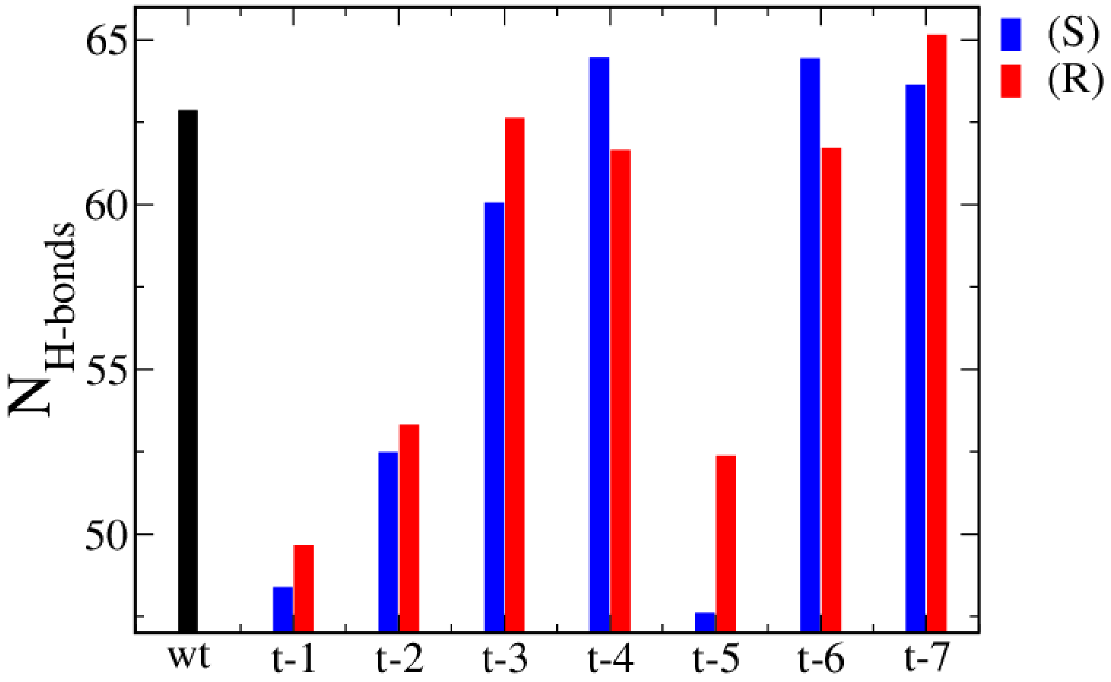

Figure S2. The number of H-bonds for each primer system. The blue and red bars correspond to the results of S-MPTE/Taq and R-MPTE/Taq complexes, respectively,

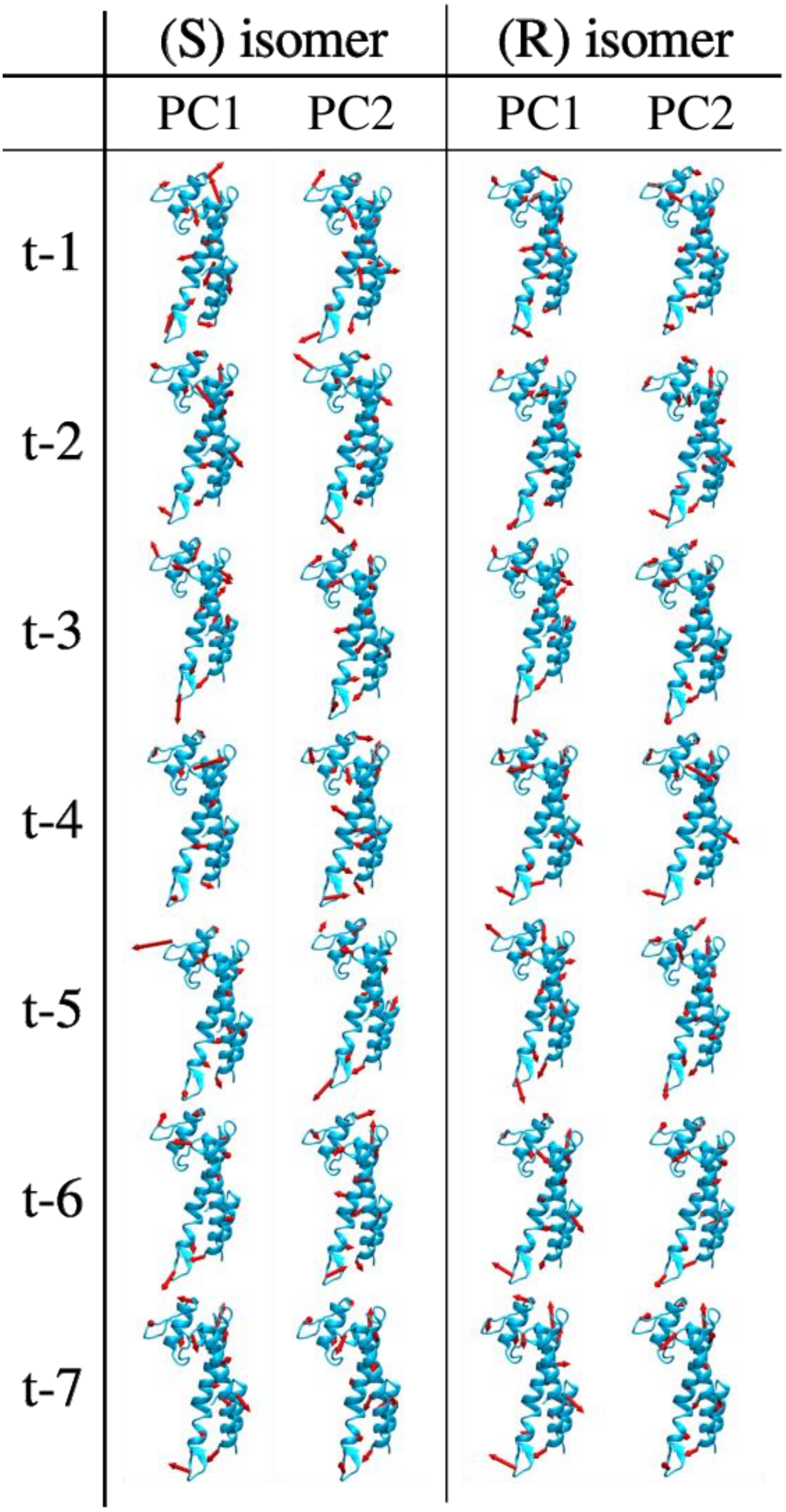

Figure S3. The motions corresponding to the two largest principal components (PCs) of thumb domain for all tested MPTE-primer systems.
